# Association between binge drinking, type of friends and gender: A cross-sectional study among Brazilian adolescents

**DOI:** 10.1186/1471-2458-12-257

**Published:** 2012-05-18

**Authors:** Patrícia M Zarzar, Kelly O Jorge, Tuula Oksanen, Miriam P Vale, Efigênia F Ferreira, Ichiro Kawachi

**Affiliations:** 1 Department of Pediatric Dentistry and Orthodontics, Faculty of Dentistry, Universidade Federal de Minas Gerais, Av. Antonio Carlos, 6627, CEP: 31270901, Belo, Horizonte/MG, Brazil; 2 Department of Society, Human Development, and Health, Harvard School of Public Health, 677 Huntington Avenue, Boston, MA, 02115-6096, USA; 3 Department of Oral Public Health, Faculty of Dentistry, Universidade Federal de Minas Gerais, Av. Antonio Carlos, 6627, CEP: 31270901, Belo, Horizonte/MG, Brazil

**Keywords:** Alcohol drinking, Friendship, Adolescent behavior, Socioeconomic factors, Epidemiology

## Abstract

**Background:**

Hazardous drinking among adolescents is a major public health concern. The purpose of this study was to examine the prevalence of binge drinking/alcohol consumption and its association with different types of friendship networks, gender and socioeconomic status among students in Belo Horizonte, Minas Gerais, Brazil.

**Methods:**

We conducted a cross-sectional study on a representative random sample of 891 adolescents (41% male, aged 15–19 years) from public and private schools in 2009–2010. Information on friendship networks and binge drinking was collected using two validated self-administered questionnaires: the Integrated Questionnaire for the Measurement of Social Capital and the first 3 items in the Alcohol Use Disorders Identification Test (AUDIT C). We used the area-based Social Vulnerability Index (SVI), mother and father’s educational background, and the type of school to assess socioeconomic status. The chi-squared test was used to examine the associations between sample characteristics or the type of friends and binge drinking (p-values <0.05 were considered statistically significant). Ordinal logistic regression was used to estimate the association between binge drinking and the independent variables.

**Results:**

A total of 321 (36%) adolescents reported binge drinking (5 or more drinks in one occasion), and among them, 233 (26.2%) adolescents reported binge drinking less than monthly to monthly, and 88 (9.9%) weekly to daily. Binge drinking was associated with being male (OR = 1.52, 95% CI 1.01–2.28) and with living in a low vulnerability area (having the best housing conditions, schooling, income, jobs, legal assistance and health) (OR = 1.66, 95% CI 1.05–2.62). Students who reported that their closest friends were from school (as opposed to friends from church) had an increased risk of binge drinking (OR = 3.55, 95% CI 1.91–5.87). In analyses stratified by gender, the association was significant only among the female students.

**Conclusions:**

The prevalence of binge drinking was high in this sample of Brazilian adolescents, and gender, low social vulnerability and friendship network were associated with binge drinking.

## Background

Binge drinking among adolescents continues to be a significant public health concern due to its high prevalence in several countries, including Thailand (33.0.%), the United Kingdom (47.0%), Germany (26.9%), Sweden (30.8%), and Brazil (34.5%) [1-5]. In Brazil, alcohol use and binge drinking represent important public health challenges because adolescents can easily purchase alcohol even though it is prohibited by law [6]. Binge drinking among adolescents is associated with the disruption of brain development [7], increased risk of alcoholism in adulthood, and increased risks for cardiovascular disease [8], disability and death [9].

Binge drinking is usually performed in groups; therefore, peers play a role in promoting binge drinking, perhaps due to peer selection or peer influence (socialization). Binge drinking has been associated with characteristics of the adolescents’ friendship networks [10-13]. Over the course of a lifetime, friendships can direct development through support, modeling, and assistance, but the significance of friendships is heightened during adolescence [14]. Peers have the potential to exert either positive or negative influences on behavior, depending on the behavior endorsed by the peer group [15,16]. During adolescence, schools may set the standards for the behavioral patterns and attitudes of the youths [17,18], and thus, students may have shared influences on their health and health-related behaviors [18]. Given the social nature of this behavior, it is not surprising that some previous work has identified associations between binge drinking and the social context, such as schools, family, and churches [10,11,13,15,19-21]. Social network effects may have both positive and negative health implications for alcohol consumption patterns, depending on the context and the circumstances [15].

Despite the preponderance of research examining the influence of peer relationships on binge drinking, few studies have evaluated the link between drinking behavior and the specific types of social relationships represented by peers. In other words, not all friendship ties are equally influential in shaping adolescent behaviors, and the aim of our study was to distinguish between the different types of friendships in various contexts [10,11,13,15,19-21].

Some of these studies [10,11,13] examined the influence of the peer group on alcohol consumption in adolescents, but they were limited to investigating just the school based peer network. However, since the influence of peer networks is not necessarily limited to the school setting, it is important that the multiple friendship networks be investigated, including those based on relationship in groups such as the church or participation in sports [19]. Understanding the relative influence of different types of peer networks on adolescent behavior can be a valuable starting point in developing interventions to modify adolescent behavior such alcohol consumption. Indeed it is more effective sometimes to intervene at the level of peer group rather than the individual, since the group can reinforce any health promotion message and provide social support for maintaining the new behavior [19].

In this paper, we examined the prevalence of binge drinking/alcohol consumption and its association with different types of friendship networks in different contexts (school, home, church, hobby groups) among male and female students in Belo Horizonte, Minas Gerais, Brazil.

## Methods

### Study design and participants

We designed a cross-sectional study to examine social and friendship networks and binge drinking among 936 adolescents aged 15 to 19 years attending public schools (n = 717, 81%) and private schools (n = 174, 19%) in the city of Belo Horizonte between August 2009 and February 2010. Belo Horizonte is the state capital of Minas Gerais, Brazil, and it has approximately two million inhabitants and is geographically divided into nine administrative districts (Brazilian Institute of Geography and Statistics/IBGE) [22]. A sample of 936 adolescents was randomly selected using a two stage stratified cluster sampling approach. The first stage was the random selection of eighteen schools (9 public and 9 private schools) in the 9 administrative districts. The second stage was the random selection of 34 classrooms within the schools. The sample size was calculated to yield a standard error of 4.0%. To calculate the required sample size for our survey, we used a 50% prevalence of alcohol consumption, which was based on statistical considerations. We used the rule suggested by others [23,24] of assuming a prior prevalence of 50% to calculate the same size.

The minimum sample size needed to satisfy the requirements was estimated to be 600 individuals. To minimize the potential losses during data collection, the sample was increased by 20% (n = 720). A design effect of 1.3 was applied to increase the precision because a multistage sampling method rather than random sampling was adopted [25]. Thus, the sample comprised 936 adolescents.

### Survey administration

*S*elf-administered questionnaires were distributed in the classroom by a researcher and assistant who collected the response envelopes immediately after being the questionnaires were completed. They explained that the responses would remain anonymous and be treated confidentially. To guard against biases that might occur due to variability in reading proficiencies, the principal investigator (K.O.J.) read aloud each question. The students could refuse to participate and return the unfilled questionnaires inside the envelopes. Among those who submitted envelopes, 4.8% of the participants (45 students) had either refused to participate or had submitted incomplete questionnaires (with 891 adolescents remaining in the study)

### Assessment of alcohol consumption

For this study, the AUDIT C (the first 3 questions of the AUDIT instrument that were related to the frequency and amount of alcohol consumed) was used because it could be employed as a stand-alone screening measure to detect hazardous drinkers among adolescents [26,27]. The Alcohol Use Disorders Identification Test (AUDIT) is a frequently used simple method for screening individuals to determine whether they were consuming excessive amounts of alcohol [26,28]. This questionnaire had been validated in Brazil [29] and had been used in a representative sample aged 15 years and over in two southeastern cities of Brazil [30,31]. This screening tool helps to identify whether an individual exhibits hazardous (or risky) drinking, harmful drinking or alcohol dependence. Binge drinking was defined as 5 or more drinks on one occasion [5]. One drink represents, on average, a can of beer or 350 ml of draft beer or 90 ml of wine, or 30 ml of a distilled beverage. Each drink contains approximately 10–12 g of alcohol [32]. The survey respondents were shown pictures of standard portions of different types of alcoholic beverages (beer, wine, whisky, and “cachaça”, a popular distilled spirit in Brazil).

We assessed binge drinking using the question “How often do you have five or more drinks on one occasion?” The response options were: Never, Less than monthly, Monthly, Weekly, and Daily or almost daily. Those who answered “Never” were coded as 0 in the analysis, “Less than monthly” and “Monthly” were coded as 1, and those who reported “Weekly” and “Daily or almost daily” frequency were coded as 2. Because binge drinking may result in more negative consequences than low to moderate drinking [33], we used binge drinking as our dependent variable.

The remaining items on the AUDIT C instrument were as follows: a) “How often did you have a drink containing alcohol in the past year?” The response options for the frequency of alcohol consumption were: “Never”, “Monthly or less”, “2 to 4 times a month”, “2 to 3 times a week”, and “4 or more times a week”; and b) “How many drinks containing alcohol did you have on a typical day when you were drinking?” The response options for the amount of alcohol consumption were “1 or 2”, “3 or 4”, “5 or 6”, “7, 8, or 9”, and “10 or more”.

### Assessment of social and friendship networks

The Integrated Questionnaire for the Measurement of Social Capital (CS-IQ) is a validated questionnaire developed by the World Bank in 2003 to assess social capital [34]. The instrument consists of 27 questions that are divided into 6 domains (groups and networks, trust and solidarity, collective action and cooperation, information and communication, cohesion and social inclusion, and empowerment and political action) [34]. The CS-QI domain selected for this study was the groups and networks domain because the study objective was to assess social behaviors related to membership in adolescent peer networks. This questionnaire was applied in a pilot test, and the students did not suggest any changes to the questionnaire. The respondents were asked to identify their most important group of close friends, and the answers were aggregated according to the most frequent response: friends from school, family, church and hobbies or other activities (sports, theatre, dances and music activities). The adolescents were also asked whether the majority of their friends’ had the same occupation and educational background. Respondents were asked about the number of groups of friends they had, and the answers were dichotomized as less than 2, and 2 or more groups of friends.

### Assessment of socioeconomic status (SES)

The Social Vulnerability Index (SVI) was used to assess the residential socioeconomic status. This index measures social exclusion in the city of Belo Horizonte. The City Hall database of SVI scores for each district was used based on the address of each student (derived from the survey) [35].

According to the theoretical framework that supported the development of the SVI, this index has 20 variables grouped into five “dimensions of citizenship”: (a) access to housing and basic infrastructure, (b) access to education, (c) access to income and employment, (d) access to legal assistance and (e) access to health, food security and welfare (35). The index categorizes residential areas into 5 classes (class I to class V), with class I indicating the highest degree of social vulnerability (the worst housing conditions, schooling, income, jobs, legal assistance and health) and class V indicating the lowest degree of social vulnerability (most advantageous conditions) [31,35-41]. The SVI has been standardized for use in characterizing area-level socioeconomic status in Brazil in several studies.

The mother and father’s educational attainment were chosen as an indicator of individual socioeconomic status because of its association to alcohol consumption/binge drinking in adolescents [42-44]. The level of education was derived from the students’ survey responses regarding the years of schooling their mothers and fathers had received. The respondents who reported that their mothers and fathers had studied for a period of between 0 to 7 years were coded as 0, and those who reported 8 or more years were coded as 1. The cut-off threshold was the median response.

The type of school was also used as a socioeconomic indicator. Although type of school is a crude assessment, wealthy adolescents in Brazil are enrolled in private schools because most Brazilian public schools are known to have less educational resources than private ones [5]. Moreover, Brazilian national epidemiological studies and others studies developed in Brazil’s southeastern cities have used this variable as an indicator of socioeconomic status [45-49]. In a recent study, the type of school was used as a socioeconomic indicator, and the authors concluded that this variable can be useful in studies involving children in Brazil [50].

### Ethics

This study was approved by the Ethics Committee of the *Universidade Federal de Minas Gerais*. Authorization was obtained from the schools to perform the research. The participants and their parents/guardians signed the informed consent.

### Statistical analysis

The chi-squared test was used to study the associations of sample characteristics or the type of friends and binge drinking (p-values <0.05 were considered statistically significant).

We used binary logistic regression analysis to examine the association of binge drinking and the type of friends. The results were expressed as odds ratios (OR) and 95% confidence intervals (CI). Each type of most important group of friends was compared to friends from hobbies/other in relation to binge drinking at frequencies ranging from less than monthly to monthly and also from weekly to daily.

An ordinal logistic regression model was used because our dependent variable was ordinal (the proportional odds model – logit link function). This model compares the likelihood of a response equal or less than a certain category with the likelihood of a highest response based on the odds ratio calculation [51,52]. The inclusion criterion was a *p* value less than 0.20 in the results of the bivariate analysis. The final model included all variables regardless of their *p*-values. Declines Homogeneity test and multi-colinearity test was performed with Pearson adjustments to analyze the validity of the final model. Because some studies have suggested that peer drinking has a greater impact on females than males [53,54], we conducted additional ordinal logistic regression models stratified by gender using the Statistical Package for the Social Sciences (SPSS for Windows, version 17.0, SPSS Inc., Chicago, IL, USA). The ordinal logistic regression models were controlled for age, gender, type of school, mother and father’s educational background and SVI.

## Results

The frequency of alcohol consumption was as follows: 212 (23.8%) adolescents reported consuming alcoholic beverages monthly or less frequently, 154 (17.3%) consumed alcohol 2 to 4 times a month, and 82 (9.2% ) adolescents reported drinking alcohol 2 or more times per week. Overall, the prevalence of any alcohol use during the past year was 50.3% (N = 448) in the sample of 16–19 year old adolescents.

The amount of alcoholic beverages consumed in a typical day when drinking alcohol was as follows: 123 (13.8%) participants reported drinking 2 to 8 or more drinks. Binge drinking was reported by 321 (36%) adolescents; of these adolescents, 233 (26.2%) reported binge drinking less than monthly to monthly and 88 (9.9%) reported binge drinking weekly to daily.

Table 1 shows the distribution of the sample based on the prevalence of the friendship network and by background characteristics. The majority of adolescents who lived in more vulnerable areas (Classes I and II) reported that the majority of their group of friends/close friends were from church, while those who lived in less vulnerable areas (Classes IV and V) reported that their most important group of close friends were from family and school networks (p < 0.001). Adolescents who reported that the majority of their groups of close friends were from church had mothers and fathers whose educational background was less than 8 years of study (p < 0.001).

**Table 1:** Distribution of the sample based on the prevalence of friendship network and by background characteristics in Belo Horizonte, Minas Gerais, Brazil, 2010 (N = 891)

									
		**School**	**Family**	Hobbies and others **	**Church**		**Total** **n (%)**	*p-value	**Degrees of freedom**
		**n (%)**	**n (%)**	**n (%)**	**n (%)**				
**Binge drinking**	Never	98 (24.7)	92 (23.2)	64 (16.2)	142 (35.9)	396 (100)			
	Less than monthly to monthly	74 (42.0)	42 (23.9)	26 (14.8)	34 (19.3)	176 (100)	28.978	0.000	6
	Weekly to daily	29 (42.6)	15 (22.1)	11 (16.2)	13 (19.1)	68 (100)			
	Total	201 (31.4)	149 (23.3)	101 (15.8)	189 (29.5)	640 (100)			
**Age**	15 to 16 years old	109 (27.6)	104 (26.3)	63 (15.9)	119 (30.1)	395 (100)		0.065	6
	17 years old	64 (38.3)	34 (20.4)	22 (13.2)	47 (28.1)	167 (100)	11.873		
	18 to 19 years old	28 (35.9)	11 (14.1)	16 (20.5)	23 (29.5)	78 (100)			
	Total	201 (31.4)	149 (23.3)	101 (15.8)	189 (29.5)	640 (100)			
**Gender**	Male	71 (31.8)	46 (20.6)	48 (21.5)	58 (26.0)	223 (100)	9.647	0.022	3
	Female	130 (31.2)	103 (24.7)	53 (12.7)	131 (31.4)	417 (100)			
	Total	201 (31.4)	149 (23.3)	101 (15.8)	189 (29.5)	640 (100)			
**Type of School**	Public	153 (31.2)	84 (17.1)	79 (16.1)	175 (35.6)	491 (100)	61.354	0.000	3
	Private	48 (32.2)	65 (43.6)	22 (14.8)	14 (9.4)	149 (100)			
	Total	201 (31.4)	149 (23.3)	101 (15.8)	189 (29.5)	640 (100)			
**Mother’s educational background**	O to 7 years of study	64 (31.4)	24 (11.8)	28 (13.7)	88 (43.1)	204 (100)	40.156	0.000	3
	8 or more years of study	106 (31.7)	106 (31.7)	49 (14.7)	73 (21.9)	334 (100)			
	Total	170 (31.6)	130 (24.2)	77 (14.3)	161 (29.9)	538 (100)			
	O to 7 years of study	66 (29.9)	35 (15.8)	31 (14.0)	89 (40.3)	221 (100)	36.996	0.000	3
**Father’s educational background**	8 or more years of study	96 (35.2)	90 (33.0)	38 (13.9)	49 (17.9)	273 (100)			
	Total	162 (32.8)	125 (25.3)	69 (14.0)	138 (27.9)	494 (100)			
	High vulnerability (class I and II)	90 (29.3)	38 (12.4)	57 (18.6)	122 (39.7)	307 (100)	54.672	0.000	3
**SVI**	Low vulnerability (class III, IV and V)	111 (33.3)	111 (33.3)	44 (13.2)	67 (20.1)	333 (100)			
	Total	201 (31.4)	149 (23.3)	101 (15.8)	189 (29.5)	640 (100)			

*Chi-squared test.

**Hobbies (friends from theater, dances, music activities, sports, and others).

Note: the total number may differ due to missing values.

Table 2 shows the association between binge drinking (less than monthly to monthly and weekly to daily) and each type of most important groups of close friends compared with friends from hobbies/other friends. Compared with those who had their most important groups of friends from hobbies and other activities, the adolescents females who reported that their most important groups of friends were from school were 2.75 times more likely to be binge drinking weekly to daily (OR = 2.75, 95% CI 1.36–5.53), and 2.99 times more likely to be binge drinking less than monthly to monthly (OR = 2.99, 95% CI 1.87–4.78). The association was not significant among the adolescent males for binge drinking weekly to daily (OR = 1.64, 95% CI 0.72–3.74) and binge drinking less than monthly to monthly (OR = 1.26, 95% CI 0.66–2.39). The female participants who reported that their most important groups of close friends were from church (as opposed to friends from hobbies/other activities) were 0.30 less likely to be binge drinking weekly to daily (OR = 0.30, 95% CI 0.12–0.73) and 0.37 less likely to be binge drinking less than monthly to monthly (OR = 0.37, 95% CI 0.22–0.63). This association was not significant among the male participants for binge drinking weekly to daily (OR = 0.72, 95% CI 0.29–1.82) nor for binge drinking less than monthly to monthly (OR = 0.59, 95% CI 0.29–1.21).

**Table 2:** Binary logistic regression of the binge drinking frequency of “weekly to daily” and “less than monthly to monthly,” and the type of friends among adolescents of Belo Horizonte, Minas Gerais, Brazil, 2010

						
**All participants**	**N of cases (%)**	**Odds Ratio (95% CI)**	**p-value**	**N of cases (%)**	**Odds Ratio (95% CI)**	**p-value**
Friends from school vs. Hobbies/others*	14.4 8.9	2.26 (1.33–3.85)	0.003	36.8 (23.2)	2.21 (1.51–3.21)	<0.001
Friends from family vs. hobbies/others*	10.1 10.8	0.94 (0.50–1.74)	0.832	28.2 (27.3)	1.04 (0.68–1.57)	0.869
Friends from church vs. Hobbies/others*	6.9 12.2	0.42 (0.22–0.80)	0.008	18.0 (31.5)	0.43 (0.28–0.66)	<0.001
Friends from hobbies vs. Hobbies/others*	10.9 10.6	1.00 (0.50–2.01)	0.651	25.7 (27.8)	0.90 (0.55–1.47)	0.358
**Females**						
Friends from school vs. Hobbies/others*	13.1 7.3	2.75 (1.36–5.53)	0.005	40.0 (20.6)	2.99 (1.87–4.78)	<0.001
Friends from family vs. Hobbies/others*	8.7 9.2	0.97 (0.44–2.15)	0.939	28.2 (26.1)	1.10 (0.67–1.83)	0.701
Friends from church vs. Hobbies/others*	4.6 11.2	0.30 (0.12–0.73)	0.008	16.0 (31.5)	0.37 (0.22–0.63)	<0.001
Friends from hobbies/others vs. Hobbies/others*	11.3 8.8	1.13 (0.44–2.90)	0.791	17.0 (28.0)	0.53 (0.25–1.15)	0.107
**Males**						
Friends from school vs. Hobbies/others*	16.9 11.8	1.64 (0.72–3.74)	0.240	31.0 (28.3)	1.26 (0.66–2.39)	0.482
Friends from family vs. Hobbies/others*	13.0 13.6	0.94 (0.35–2.52)	0.894	28.3 (29.4)	0.94 (0.45–1.96)	0.859
Friends from church vs. Hobbies/others*	12.1 13.9	0.72 (0.29–1.82)	0.489	22.4 (31.5)	0.59 (0.29–1.21)	0.152
Friends from hobbies vs. Hobbies/others*	10.4 14.3	0.78 (0.27–2.25)	0.651	35.4 (27.4)	1.39 (0.69–2.80)	0.358

* Hobbies/others (friends from theater, dances, music activities, sports, and others).

Table 3 shows the association between binge drinking and friendship networks. The adolescents who reported that the most important groups of close friends were from school (as opposed to friends from church) had a higher likelihood of binge drinking (OR = 3.55, 95% CI 1.91–5.87). In analyses stratified by gender, the association was significant only among the female participants (OR = 3.70, 95% CI 1.84–7.45). The adolescent males who reported having more than 2 groups of friends had increased odds of binge drinking (adjusted OR = 2.21, 95% CI 1.00–4.87) compared with those who reported having less than 2 groups of friends. Adolescents from low vulnerability areas (best social and economic conditions) had 1.66 times higher odds of binge drinking (adjusted OR = 1.66, 95% CI 1.05–2.62).

**Table 3:** Ordinal logistic regression of binge drinking and friendship networks, comparing binge drinking frequency of “less than monthly to monthly” frequency with “ weekly to daily” with “never” binge drinking among adolescents in Belo Horizonte, Minas Gerais, Brazil (n = 891), 2010

										
		**Crude OR (95% CI)**	**Adjusted OR (95% CI)**	p-value Adjusted*	**Crude OR (95% CI)**	**Adjusted OR (95% CI)**	p-value Adjusted*	**Crude OR (95% CI)**	**Adjusted OR (95% CI)**	p-value Adjusted*
**Most Important groups of friends or close friends**	School	3.77 (2.27–6.25)	3.55 (1.91–5.87)	<0.001	4.25 (2.25–8.03)	3.70 (1.84–7.45)	<0.001	2.91 (1.24–6.84)	2.44 (0.91–6.46)	0.073
	Family	1.99 (1.16–3.43)	1.79 (0.98–3.27)	0.055	2.41 (1.24–4.72)	2.00 (0.96–4.17)	0.064	1.34 (0.52–3.44)	1.49 (0.50–4.50)	0.474
	**Hobbies/ others	1.89 (1.00–3.57)	1.54 (0.78–3.04)	0.207	2.11 (0.89–4.96)	1.74 (0.71–4.29)	0.225	1.30 (0.49–3.45)	1.12 (0.37–3.35)	0.837
	Church	1.00	1.00		1.00	1.00		1.00	1.00	
**Majority of friends that have the same work or occupation**	Yes	1.57 (1.08–2.29)	1.25 (0.79–1.98)	0.340	1.60 (1.00–2.54)	1.20 (0.67–2.16)	0.538	1.50 (0.80–2.83)	1.31 (0.59–2.94)	0.505
	No	1.00	1.00		1.00	1.00		1.00	1.00	
**Educational background or level for the majority of friends**	Yes	1.40 (0.94–2.08)	0.73 (0.44–1.18)	0.200	1.53 (0.93–2.51)	0.80 (0.43–1.50)	0.492	1.17 (0.59–2.31)	0.63 (0.27–1.46)	0.282
	No	1.00	1.00		1.00	1.00		1.00		
**Number of groups of friends**	< 2	1.25 (0.84–1.85)	1.37 (0.89–2.11)	0.143	1.01 (0.62–1.65)	1.14 (0.68–1.93)	0.618	2.15 (1.06–4.36)	2.21 (1.00–4.87)	0.049
	≥ 2	1.00	1.00		1.00			1.00	1.00	
**Age**	15–16	0.98 (0.65–1.49)	0.82 (0.44–1.52)	0.524	0.84 (0.36–1.95)	0.66 (0.27–1.59)	0.358	0.95 (0.41–2.20)	0.88 (0.35–2.21)	0.793
	17	1.17 (0.74–1.84)	1.48 (0.76–2.86)	0.245	1.31 (0.54–3.19)	1.01 (0.40–2.54)	0.974	2.22 (0.87–5.71)	2.44 (0.90–6.63)	0.081
	18-19	1.00	1.00		1.00					
**Mother’s educational background**	0–7 years	0.66 (0.45–0.98)	0.94 (0.56–1.58)	0.825	0.63 (0.39–1.01)	1.01 (0.52–1.95)	0.970	0.78 (0.40–1.51)	0.87 (0.36–2.11)	
	8 or more	1.00	1.00		1.00	1.00		1.00	1.00	
**Father’s educational background**	0–7 years	0.60 (0.43–0.83)	1.08 (0.65–1.80)	0.751	0.61 (0.38–0.97)	0.93 (0.48–1.79)	0.830	0.88 (0.47–1.63)	1.47 (0.63–3.43)	0.376
	8 or more	1.00	1.00		1.00	1.00		1.00	1.00	
**Type of school**	Public	0.60 (0.40–0.89)	0.74 (0.45–1.23)	0.245	0.59 (0.36–0.96)	0.80 (0.43–1.49)	0.485	0.64 (0.33–1.25)	0.63 (0.30–1.35)	0.236
	Private	1.00	1.00		1.00	1.00		1.00	1.00	
**IVS**	Low Vulnerability	1.66 (1.15–2.41)	1.66 (1.05–2.62)	0.028	1.83 (1.46–2.92)	1.62 (0.90–2.92)	0.104	1.48 (0.80–2.76)	1.97 (0.90–4.32)	0.089
	Great Vulnerability	1.00	1.00		1.00	1.00		1.00	1.00	
**Gender**	Male	1.24 (0.94–1.62)	1.52 (1.01–2.28)	0.044						
	Female	1.00	1.00							

* Adjusted for age, sex, type of school, mother’s education and Social Vulnerability Index (SVI).

**Hobbies/others (friends from theater, dances, music activities, sports, and others).

## Discussion

In a random sample of 891 Brazilian adolescents 15 to 19 years of age, half of the participants reported alcohol consumption during the past year. One-third of the participants reported binge drinking. Having their most important groups of friends from school was associated with a higher likelihood of binge drinking. Individual SES was not associated with binge drinking, but residential SES was associated with binge drinking.

The prevalence of alcohol consumption in our study in Belo Horizonte, Brazil, was similar to previous studies conducted among adolescents in Brazil [5,56,57]. However, some studies have reported lower prevalence compared with the present survey [32,58]. The prevalence of binge drinking in the present study (36%) was similar to that reported by studies from other countries (27%–47%), and our results are comparable to figures reported previously in Brazil (35%) [5]. Although the comparison of binge drinking prevalence between countries is somewhat complicated by differences in standard drink sizes and other definitions, the high rates found for alcohol consumption among adolescents represent a significant public health concern. Binge drinking was associated with being an adolescent male, and this result corroborates findings in previous studies [5,56].

Some studies of alcohol consumption and binge drinking in adolescents have shown a statistically significant association with socioeconomic status (family income, personal income and unemployment), but conflicting results have also been reported in the literature. Some studies confirmed that alcohol consumption is negatively associated with socioeconomic status [59,60], while others found positive associations between higher socioeconomic status and high-risk drinking [56,61] as well as low socioeconomic conditions and high-risk drinking [30,32]. Consistent with other studies [55,59-61], our results indicate a higher prevalence of binge drinking among adolescents who live in less vulnerable areas. Also consistent with our results, Humensky (2010) [61] indicated that a higher socioeconomic status in adolescence, as measured by parental education and household income, is associated with higher rates of substance use, particularly binge drinking. Because the SVI also includes family income and educational backgrounds, these results consider the disposable income of those who live in less vulnerable areas where adolescents are more likely to buy their own alcohol and consume it at rates and in quantities without guidance. According to a longitudinal study developed in a representative sample of adolescents in Sweden with multilevel analysis of a period with large economic changes, unemployment is statistically associated with binge drinking [3]. A decrease in incomes leads to lower consumption of all goods, including alcohol because adolescents have to allocate the resources they can spend on different goods [3].

In this paper, we estimated the predictors of adolescent drinking behavior to identify the role of social friendship networks on binge drinking. Adolescents who reported that the majority of their groups of friends/close friends were from school rather than from church or hobbies/other activities had higher odds of binge drinking. This result corroborates finding in the National Longitudinal Study of Adolescent Health in the US, suggesting that an increase of 10% in the proportion of classmates who drink will increase the likelihood of drinking among peers by 4% [12]. With similar results, a classroom-randomized controlled trial that compared control classes with those receiving an evidence-based substance use (including alcohol) prevention program with 541 adolescents in the US indicated that students with classroom friends who used substances were more likely to increase their own use [10]. However, our study revealed that differences emerged when analyses were conducted separately by gender. In our study, the adolescent females who reported that the majority of their groups of close friends were from school had a higher likelihood of binge drinking compared with those who reported that their best friends were from church or from hobbies and other activities. However, no statistically significant association was found for male adolescents. These results are consistent with previous evidence of the importance of friends on alcohol drinking in adolescence. At least two studies have demonstrated that peer drinking has a greater impact on the average amount of alcohol consumed by girls consumed versus the amount consumed by boys [53,62]. Furthermore, girls are more likely to socialize with and be persuaded by friends to drink alcohol compared with boys [55].

When we compared the adolescents who reported that the majority of their groups of close friends were from church with the adolescents who reported that the majority of their groups of close friends were from hobbies and other activities, we found that having friends from church was associated with less binge drinking. This result is similar to that found using other surveys, which suggest the association between attending church/religiosity and a lower likelihood of binge drinking [5,20,63,64]. However, because we did not collect information on the adolescents’ religious beliefs or church attendance frequency, we were unable to tease out the relative contributions of having friends in church-based networks versus religious participation or religious beliefs.

Studies developed by Valente et al. (2007) [10]; Jamison and Myers (2008) [11]; Kiuru et al. (2010) [13], previously examined the influence of the peer group on alcohol consumption in adolescents, but they were limited to investigating just the school based peer network. Other studies investigated the influence of the peer group on alcohol consumption in adolescents based on their religiosity or church attendance [5,63]. We are not aware of a previous study that compared the different type of groups of friends from different contexts in relation to alcohol consumption by adolescents. It is important to consider friendship networks in multiple contexts when examining peer influences on adolescent behavior. Our study was not restricted to nominated peer groups, so it was possible to compare groups of friends from different contexts such as church, school, family and hobby groups. Comparing the different sources of peer connections, we found that adolescent binge drinking was strongly associated with school-based friendship networks relative to church-based networks.

Throughout the course of a lifetime, friendships can direct development through support, modeling, and assistance, but their significance is heightened during adolescence [14]. Peers have the potential to both positively and negatively influence behavior, depending on the behavior endorsed by the peer group [15].

Public health interventions targeted to the peer network might be more cost-effective than previous estimates have suggested because the health-promoting behavior of one person may spread to others via social networks [10,12]. The extent to which the school is a functional community with supportive social relationships, social participations in school activities, and shared norms, goals, and values may also moderate the individual risk of initiating adverse health behaviors such as high alcohol consumption [65]. Our results showed that students having their most important group of friends coming from church seems to be a protective factor with respect to binge drinking. The protective effect of social capital might reflect the effect of norms and social controls on curtailing deviant and dangerous alcohol consumption in communities in which individuals are more bonded to each other [66]. Thus, our findings underscore the importance of deeply exploring how the context can determine alcohol consumption.

`Our results should be interpreted while considering some limitations. Because this study of the association between type of friendship network and binge drinking used cross-sectional data, two principal threats to causal inference should be considered: homophily and unobserved confounding. Homophily refers to the idea that individuals select different types of social network connections based partly on their own behavior preferences. For example, some students may enjoy associating with others based on their shared preference for observing religious proscriptions against drinking alcohol. In other words, the associations that we observed may have been driven by the tendency for “birds of a feather to flock together”, rather than by the network characteristics per se (e.g., social norms that influence and regulate behaviors within certain types of social networks). Secondly, our finding may reflect the influence of unmeasured confounding factors that affect both patterns of friendship networks and drinking behavior. For example, the strictness of school policies against drinking may influence both the formation of different friendship connections as well as the prevalence of binge drinking. Because we did not collect information on variables such as school policies, we cannot rule out the possibility that unmeasured or unobserved factors could have led to the patterns we observed.

We did not measure and control for variables such as “parental supervision” in our survey. Thus, if students who associated with peers from school were also those who were less likely to be supervised by their parents, the association between having school friends and binge drinking could have been confounded by such omitted variables.

We assured the participants in this study that their responses would remain anonymous. However, there are some limitations associated with our measures of behaviors and negative outcomes because they rely on self-reports of alcohol consumption and binge drinking. It is possible that the actual amount of alcohol consumption and binge drinking could have been either under-reported or exaggerated. Furthermore, our sampling approach did not include individuals who were excluded from or had otherwise left school-based education, and the Vulnerability Index (SVI) was calculated on an ecologic level and not on an individual basis.

The level of social capital to which an individual may gain access through their social networks depends on the structural characteristics of the networks as well as on the amount of social capital that other individuals in the network possess. Although they may be positive, friendship networks can also be viewed as negative, especially with regard to binge drinking during adolescence. Corroborating our results, at least three other studies have also found an association between peer influence and alcohol consumption and binge drinking [10,11,13].

We were unable to obtain information on the size, density, quality, or proximity of the friendship network or the normative drinking behaviors within those networks. Further studies are needed to investigate the importance of the detailed characteristics of friendship networks (size, density, quality, and proximity), parental control, religious beliefs and participation. Another important issue for future research is to measure the perceived norms among different types of friendships. Ideally, studies should measure the subjective norms surrounding drinking behavior within school-based versus church-based networks (e.g., “How frequently do my friends from school/church engage in binge drinking?” and “Do my friends (in school or in my church group) approve/disapprove of drinking?”).

## Conclusions

The findings of this study highlight the association between social network (type of friends) and risky drinking behavior among adolescents, the importance of which is underscored in countries like Brazil that have a high prevalence of alcohol consumption and binge drinking among adolescents. This study revealed that the type of group of friends with which an adolescent associates and gender may determine the likelihood that an individual consumes large amounts of alcohol. Therefore, interventions to reduce alcohol consumption in adolescents should consider both the friendship network characteristics and gender.

## Competing interests

The authors declare that they have no competing interests.

## Authors’ contributions

PZ, KJ, MV, and EF conceived of the study. KJ collected data. PZ conducted the analysis and wrote the first version. TO and IK contributed substantially to the interpretation of the results. KJ, TO, MV, EF and IK revised the manuscript for important intellectual content. All authors read and approved the final version of the manuscript.

## Pre-publication history

The pre-publication history for this paper can be accessed here:

http://www.biomedcentral.com/1471-2458/12/257/prepub
